# The Use of E-mail in Primary Care: Characterization of the Reality of a Family Health Unit

**DOI:** 10.7759/cureus.87974

**Published:** 2025-07-15

**Authors:** Adriana Silva, Patricia B Nascimento, Amaro Henriques, Liliana Portela, Diogo B Almeida, Vasco Varela

**Affiliations:** 1 Family Medicine, Unidade Local de Saúde Lisboa Ocidental, Lisboa, PRT

**Keywords:** clinical governance, e-mail, healthcare digitalization, primary health care, telemedicine

## Abstract

Introduction

The progressive digitalization of healthcare has fostered the utilization of e-mail as a communication tool between users and family physicians. This modality has the potential to enhance accessibility, ensure continuity of care, and support clinical decision-making. Nevertheless, concerns persist regarding data security, equitable access, and increased workload for professionals. This study aims to characterize the use of e-mail communication within a Family Health Unit in Lisbon.

Methods

A descriptive, observational, cross-sectional, and retrospective study was conducted, based on the analysis of 1,282 e-mails sent by users to two family physicians between July and December 2021. Variables analyzed included user characteristics (sex, age, educational attainment), reasons for contact, response rate, and time, as well as the subsequent clinical or administrative outcomes resulting from the e-mail exchanges.

Results

Among the users, 71.68% were female, with a mean age of 48.2 years. Of the 390 users for whom educational data were available, 94.10% had attained higher education. The main reasons for contact were requests for prescriptions or test results (35.34%), administrative matters (14.66%), and repeat prescription requests (11.86%). Only 32.06% of the e-mails received a response, with a median response time of 2 days. Approximately 11% of the contacts led to in-person or telephone consultations, and 26.37% were documented in the clinical record.

Conclusion

The findings reflect an increased reliance on e-mail communication in primary care, accentuated by the digital transformation following the COVID-19 pandemic. The results underscore the necessity of establishing regulatory frameworks, providing professional training, and allocating adequate resources to ensure the safe, efficient, and equitable integration of this tool into routine clinical practice.

## Introduction

Information and communication technologies, along with the internet, have been recognized as key mechanisms in the transformation of medical care, enabling the progressive reorganization of services and facilitating both accessibility and continuity of health care [1]. In primary health care (PHC), there has been a growing interest in the implementation of digital services aimed at enhancing communication between health professionals and users, promoting faster interaction aligned with the current needs of the population [2]. Within this framework, asynchronous e-mail communication between patients and physicians has gained prominence as a complementary tool to clinical interactions [3]. These consultations are defined as online interactions that allow communication between family physicians and their patients without requiring physical and/or temporal presence, thereby reinforcing access to care [4]. They are not intended to replace face-to-face clinical encounters but rather to serve as an additional tool to support the therapeutic process.

Several studies have identified benefits associated with the use of e-mail in clinical practice, including improved access to care, the provision of more thoughtful and planned care, facilitation of the communication of sensitive issues by users, dissemination of relevant health information, and promotion of care continuity, particularly for patients with chronic conditions [5-8]. This communication method has also been associated with the strengthening of the doctor-patient relationship [7]. Nonetheless, limitations and concerns have also been acknowledged: risks related to the security and confidentiality of exchanged data [2,3,7-10], the potential digital exclusion of more vulnerable populations, such as the elderly and individuals with low digital literacy [2,7], and the burden of unpaid additional workload on professionals [2,3].

Adoption of e-mail-based communication between patients and physicians varies across Europe, reflecting the specific health policies of each country [11]. In Denmark, this modality is mandatory and accounts for approximately 21% of medical consultations [4], whereas in other contexts, such as the United Kingdom, only 6% of general practitioners report using it [7]. In Portugal, data are limited. A study conducted in the Local Health Unit of Matosinhos (2022) identified greater use among physicians working with larger patient lists. More than half reported receiving e-mails daily, and 43.2% responded frequently, although one-third did not use this communication method regularly [10].

Regarding the characterization of asynchronous e-mail communication between patients and family doctors in Portugal, available studies date back to 2009 [6,8] and 2011 [9], conducted in three Family Health Units (FHU). These studies reported that the majority of users were female [6,8], that e-mails were primarily sent during the operating hours of the FHU [8,9], and that the main topics included medical inquiries, administrative matters, test results, and medication [6,8,9].

Considering the momentum generated by the COVID-19 pandemic in promoting remote communication and the increasing digitalization of PHC, this study aims to characterize the use of e-mail communication between patients and two family physicians in a FHU in Lisbon. Specifically, it focuses on describing the demographic profile of users, the reasons for contact, physician response patterns, and the clinical or administrative outcomes resulting from these interactions.

## Materials and methods

Study design and setting

This study employed a descriptive, observational, retrospective, and cross-sectional design. It was conducted within an FHU and aimed to analyze the use of e-mail as a communication tool between patients and their family physicians. The study covered a six-month period, from July to December 2021.

Participants and selection criteria

Two family physicians from the FHU were included in the study. These professionals were selected based on their consistent and habitual use of e-mail as a means of communication with patients, as well as their willingness and availability to participate in the research. Only e-mails sent directly by patients were considered eligible for inclusion in the study. Messages received from other healthcare professionals, administrative staff, or institutional sources were excluded to ensure that the analysis focused exclusively on patient-initiated communication. Both physicians had a practice of informing patients, during face-to-face consultations, about the appropriate use and limitations of e-mail communication. This guidance was reinforced through an automatic reply system that acknowledged receipt of incoming messages and provided further clarification regarding expectations for response times and appropriate usage, including, for example, the recommendation that acute symptoms should not be communicated via e-mail due to the potential for delayed response.

Data collection and variables

Data were collected from institutional e-mail accounts associated with the physicians, hosted on the Office 365 platform. The e-mail messages were reviewed and categorized according to several variables. These included patient demographic characteristics such as age, sex, and education level. The reason for the e-mail was also recorded, encompassing categories such as prescription requests, delivery of test results, appointment scheduling, symptom reporting, COVID-19-related concerns, administrative queries, and other topics. Additionally, the nature of the physician's response was analyzed, including whether a reply was sent and the time interval between receipt and response. Finally, the study considered the clinical or administrative outcomes of each e-mail, specifically whether it resulted in a new entry in the daily agenda or the scheduling of an in-person consultation.

Duplicate e-mails were defined as messages sent more than once by the same user regarding the same issue - either following a previous reply or due to the absence of a response, or by mistake - and were excluded to avoid overrepresentation of a single request. The process of identifying and excluding duplicates was carried out manually by the research team through careful analysis of message content, sender identity, and timing. In cases where e-mails addressed multiple topics, classification was based on the predominant reason for contact, as determined by clinical relevance and message content.

To ensure a comprehensive understanding of follow-up actions, data collection was supplemented through the review of medical scheduling systems used within the FHU. These included MedicineOne and SClínico, the latter of which was implemented during the study period. Each patient’s data was reviewed only once. Importantly, the participating physicians were not aware, during the study period, that their e-mail activity would be analyzed, which contributed to a more naturalistic observation of clinical practice and minimized potential behavioral changes associated with observation.

Researchers manually reviewed each patient’s data only once.

Data analysis

Descriptive statistical analysis was conducted using Microsoft Excel®, allowing for the quantification and characterization of the communication patterns observed between patients and their physicians.

Data protection and ethical considerations

All data were anonymized and encrypted prior to analysis and were stored on secure systems to maintain confidentiality and adhere to data protection regulations. The study was approved by the Ethics Committee of the Lisbon and Tejo Valley Regional Health Administration, approval number 6229/CES/2022, in October 2022, and all procedures were conducted in accordance with the ethical standards outlined in the Declaration of Helsinki.

## Results

Between July and December 2021, a total of 3,684 e-mails were received by two family physicians working in an FHU. After applying the exclusion criteria - specifically, removing 1,985 e-mails sent by healthcare professionals or institutions, as well as 417 identified as duplicates - a final sample of 1,282 patient-initiated e-mails was included in the analysis. This corresponds to an average of approximately 107 e-mails per month, or four per day, per physician, highlighting a stable and continuous use of e-mail communication within routine clinical practice during the study period. The results are summarized in Table 1.

**Table 1:** Analysis of Patient-Initiated E-mails: Demographics, E-mail Purposes, Response Characteristics, and Resulting Outcomes

Section	Category/Parameter	Value	Percentage
E-mail Volume and Selection	Total e-mails received	3684	100
	E-mails excluded (from professionals/institutions)	1985	53.89
	Duplicate e-mails	417	11.33
	E-mails analyzed	1282	34.81
	Avg. e-mails/month/physician	107	-
	Avg. e-mails/day/physician	4	-
E-mail Users	Female users	919	71.68
	Male users	363	28.32
	Age range	17–91 years	
	Mean age	48.2 years	
	Users aged >65	155	12.09
Education Level (n=390)	Basic education	17	4.36
	Secondary education	6	1.54
	Higher education	367	94.10
Reasons For E-mails (N=1282)	Prescription/test result requests	453	35.34
	Administrative issues	188	14.66
	Prescription renewal requests	152	11.86
	COVID-19 related matters	145	11.31
	Other subjects	132	10.30
	Symptom description	113	8.81
	Request for medical consultation	99	7.72
Response Characteristics	E-mails responded to	411	32.06
	Response time range	0–38 days	
	Median response time	2 days	
Response Time Distribution	Same day	98	23.84
	Within 5 days	303	73.72
	Between 5 and 10 days	73	17.76
	More than 10 days	35	8.52
Outcomes Triggered		All E-mails (n = 1282)	Responded E-mails (n = 411)
	Registered in the schedule	338 (26.37)	201 (48.91)
	Not registered in the schedule	944 (73.63)	210 (51.09)
	No further action	1139 (88.92)	353 (85.89)
	Phone consultation	44 (3.43)	13 (3.16)
	In-person consultation	98 (7.64)	45 (10.95)

Demographic and educational profile of E-mail users

The patient population using e-mail was predominantly female, accounting for 71.68% of users, compared to 28.32% male users. Patient age ranged from 17 to 91 years, with a mean of 48.2 years (SD = 14.1). A noteworthy proportion, 12.09%, were aged 65 or older, reflecting some digital engagement among older adults. Educational background was available for 390 users (30,4%): of these, 94.10% held higher education degrees, 4.36% had completed only basic education, and 1.54% secondary education. These findings suggest that the use of e-mail as a healthcare communication tool was more prevalent among middle-aged, highly educated individuals, particularly women. Although less frequent, the participation of older adults (12% over the age of 65) still reflects some degree of digital engagement within this age group.

Nature and purpose of E-mail communication

The primary reason for e-mail contact was related to the communication of diagnostic test results, which accounted for 35.34% of all messages. This was followed by administrative requests (14.66%), medication prescription renewals (11.86%), and issues linked to COVID-19 infection (11.31%). Less frequent categories included other uncategorized topics (10.30%), symptom descriptions (8.81%), and appointment scheduling requests (7.72%). The diversity of topics suggests that patients used e-mail to address a broad range of needs, primarily those that did not require immediate clinical intervention, reinforcing its value for follow-up, administrative tasks, and general inquiries.

Physician response patterns and timeliness

Of the 1,282 e-mails analyzed, 32.06% received a response from the physicians. Response times ranged from the same day to 38 days later, with a median of two days. Among the e-mails that were answered, 23.84% were responded to on the same day, 73.72% within the first five days, 17.76% between days five and ten, and 8.52% after more than ten days. While many responses occurred within a timeframe considered reasonable for asynchronous communication, the overall response rate was low, suggesting variability in physician engagement or prioritization of messages and possible telephone consultations that were not registered in the agenda.

Impact and outcomes resulting from E-mail interactions

Out of all e-mails received, only 26.37% were formally documented in the physicians’ clinical schedule, with 73.63% lacking such registration. Among the subset of 411 responded e-mails, 48.91% were recorded, indicating a partial but not systematic integration of e-mail exchanges into clinical records. Regarding subsequent actions, the majority of e-mails (88.92%) did not result in further clinical activity. However, 3.43% prompted telephone consultations and 7.64% led to in-person consultations. Within the responded group, only 3.16% required a telephone contact, and 10.95% culminated in an in-person visit. These results emphasize that most patient concerns submitted via e-mail were appropriately addressed within the asynchronous exchange itself, with a limited proportion necessitating escalation to direct contact, which illustrates the efficiency of e-mail for handling low-complexity clinical and administrative matters.

## Discussion

This study highlighted the growing use of e-mail between patients and family physicians, with 1,282 e-mails received by two doctors between July and December 2021, averaging 107 per month and 4 per day per physician. These figures exceed those reported in previous national studies: 34 e-mails per month in the study by Pinhão et al., 36 in Granja, and 12 in the study by Ponte [6,8,9]. The time difference between studies and the pandemic context, which accelerated the digitalization of health care, may help explain this increase.

The mean age was 48.2 years, higher than that reported by Pinhão et al. (44.8) and Granja (37.1), reflecting greater engagement with digital technologies among older age groups [6,8]. In this study, 12% of users were over the age of 65, twice the proportion reported by Granja [8]. This could be explained by the demographic characteristics of the local population (18.2% with secondary and 33.9% with higher education), as well as increased digital literacy following the pandemic [12].

Regarding sex, 72% of e-mails were sent by women, consistent with other national studies [6,8,9], but contrasting with the international findings of Atherton et al., where men were the majority (56%) [7]. This may reflect the higher use of health care services among female users in Portugal [13]. Educational attainment was identified in 30% of cases, of which 94% had higher education, in line with findings from other studies [8,14].

The most frequent reason for contact was the request for diagnostic tests or the communication of their results (35%), a proportion higher than that reported by Granja (15%) and Ponte (19%) [8-9]. This discrepancy may reflect limited access to face-to-face consultations during the pandemic. Administrative matters accounted for 15%, lower than in Pinhão et al. (37%) and Ponte, possibly due to the existence of an institutional e-mail account managed by the administrative team in the FHU of the present study [6,9].

Prescription requests represented 12%, slightly below the 15% reported by Granja, which may be explained by the availability of a dedicated request form at the FHU in this study [8]. COVID-19-related issues (11%) and symptom descriptions (9%) were in line with previous studies [8,9], but higher than the 4% reported by Atherton et al. [7]. This higher frequency may be associated with the pandemic context, despite the presence of automatic replies discouraging the use of e-mail for acute situations.

Only 32% of e-mails received a reply, below the 69% reported by Granja [8]. This discrepancy may be due to the higher message volume and prior agreements with patients indicating that replies would be sent only when changes were necessary. Additionally, some issues may have been resolved administratively or by telephone, without documentation in the e-mail system or medical record.

Response times ranged from the same day to 38 days, with a median of 2 days - similar to Atherton et al., but longer than the 4-day maximum reported by Leong et al. [7,14]. This difference may be related to the lower number of e-mails and increased physician awareness in that study. In this study, 74% of replies were sent within the first five days, and 24% on the same day. Granja reported 52% within 12-48 hours, and Ponte 57% within 12 hours [8-9]. Occasional initial triage by administrative staff when e-mails were sent to the general e-mail of the FHU and clinical workload pressures may have contributed to longer response times.

Only 49% of replies were documented in the medical record, which may undermine recognition of the work performed and hinder effective planning of clinical workload. No prior studies were identified that quantified this indicator.

In terms of outcomes, 32% of e-mails received a response, 3% led to telephone consultations, and 8% to in-person consultations, totaling 43%. Granja reported 66% [8]. This difference may reflect the nature of the requests, such as prescriptions and test results. The rate of in-person consultations (8%) was lower than the 13% observed by Atherton et al. [7].

Among the strengths of this study is the analysis of the impact of digitalization in a pandemic context and the minimization of the Hawthorne effect, as physicians were unaware that they would be studied [15]. Limitations include the small number of participating physicians, the single-site focus, and the potential underestimation of e-mails resolved through undocumented means. The lack of consensus on how to categorize reasons for contact across studies also limits direct comparability. Although both participating physicians shared similar working conditions and had comparable patient lists in terms of size and demographic profile, individual characteristics - such as their digital literacy, communication preferences, and workload management - may have influenced the volume and nature of e-mail use observed. These factors were not directly assessed in the present study but should be considered in future research involving a broader and more diverse group of professionals and settings.

## Conclusions

The rise in remote interactions in PHC, particularly in the wake of the COVID-19 pandemic, has reinforced the role of e-mail as a valuable complementary tool in the doctor-patient relationship. This study, conducted in an FHU in Lisbon, demonstrated the utility of e-mail communication in addressing a broad spectrum of needs, from clinical requests to administrative matters, while also highlighting the commitment of physicians to maintaining accessibility. Notably, the majority of responses were provided within five days, despite the lack of protected time in clinical schedules for managing this type of interaction.

The findings underscore the enduring importance of the family physician in fostering proximity and continuity of care - even in digital contexts. However, as this was a descriptive, single-site study involving physicians already accustomed to using e-mail, the observations should be interpreted within their specific context and not generalized to other settings without caution. Furthermore, outcomes such as care quality, safety, and efficiency were not directly assessed, and future research may complement these findings by exploring those dimensions.

To fully harness the benefits of this communication channel, it is essential to establish a clear legal framework for telemedicine, adapt physicians’ working conditions to reflect emerging digital realities, and promote equitable access and digital literacy. These measures would help ensure the safe, equitable, and sustainable integration of e-mail communication into PHC practice.

The recommendations arising from this study are aligned with broader strategic frameworks, such as the Portuguese National Strategy for Health in the Digital Era and the WHO Global Strategy on Digital Health 2020-2025, both of which underscore the importance of regulatory guidance, digital inclusion, and the development of professional competencies to support the effective integration of digital tools into clinical care.
